# Exploiting Interdata Relationships in Next-generation Proteomics Analysis*

**DOI:** 10.1074/mcp.MR118.001246

**Published:** 2019-05-24

**Authors:** Burcu Vitrinel, Hiromi W. L. Koh, Funda Mujgan Kar, Shuvadeep Maity, Justin Rendleman, Hyungwon Choi, Christine Vogel

**Affiliations:** ‡Center for Genomics and Systems Biology, Department of Biology, New York University, New York, NY; §Department of Medicine, Yong Loo Lin School of Medicine, National University Singapore, Singapore; ¶Institute of Molecular and Cell Biology, Agency for Science, Technology, and Research, Singapore

**Keywords:** Systems biology*, Bioinformatics, Computational Biology, RNA SEQ, Modeling, Post-translational modifications*, Translation*, Transcription*, Degradomics*, Metabolomics, integration, multiomics, systems biology

## Abstract

Mass spectrometry based proteomics and other technologies have matured to enable routine quantitative, system-wide analysis of concentrations, modifications, and interactions of proteins, mRNAs, and other molecules. These studies have allowed us to move toward a new field concerned with mining information from the combination of these orthogonal data sets, perhaps called “integromics.” We highlight examples of recent studies and tools that aim at relating proteomic information to mRNAs, genetic associations, and changes in small molecules and lipids. We argue that productive data integration differs from parallel acquisition and interpretation and should move toward quantitative modeling of the relationships between the data. These relationships might be expressed by temporal information retrieved from time series experiments, rate equations to model synthesis and degradation, or networks of causal, evolutionary, physical, and other interactions. We outline steps and considerations toward such integromic studies to exploit the synergy between data sets.

## Abstract (precis)

Mass spectrometry-based proteomics and other technologies have matured to enable routine acquisition of system-wide data sets that describe concentrations, modifications, and interactions of proteins, mRNAs, and other molecules. Productive integrative studies differ from parallel data analysis by quantitative modeling of the relationships between data. We outline steps and considerations towards integromic studies to exploit the synergy between data sets.

## Graphical Abstract

**Figure:**
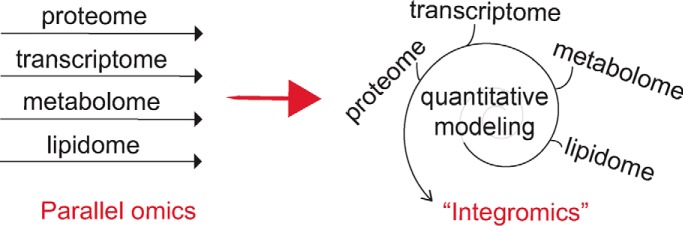

**Highlights**

- Technological advances produce many system-wide 'omics data sets.
- Integrating proteomics with other data needs quantitative modeling of inter-data relationships.
- Some tools have emerged for considering specific properties of 'omics data.
- Productive integration of proteomic and other data can provide exciting new insights.

## The past and the present: directions in proteomic data integration

**Fig. 1.**
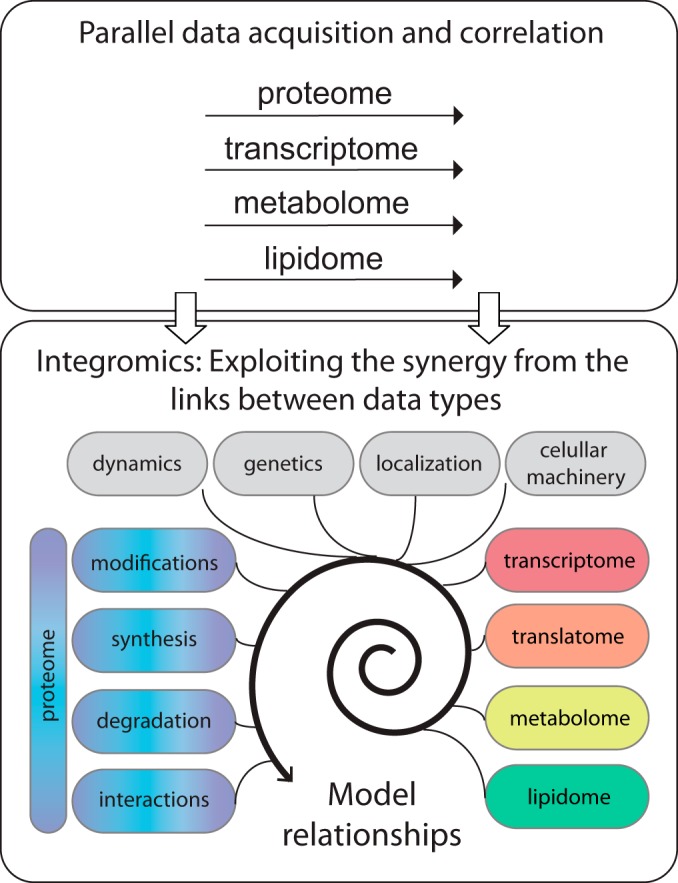
**Moving from multiomics studies that acquire and analyze data sets in parallel to modeling and exploiting the interactions between data.**

Recent large-scale analysis of protein concentrations, modifications, and interactions has seen tremendous advances, pushing us to consider the next steps in multiomics studies. Some of the new work lies “outside the box” of standard parallel mining of individual data sets and attempts to model the relationships between proteomic variations and other molecular changes to gain insights at their interface (Fig. 1). A new field might be born: “integromics.” Integromics studies have included information on the dynamics of mRNA and protein concentration changes, but also other molecules, such as lipids and metabolites, or completely orthogonal information on genomic variation across a population of samples. Because several excellent reviews discuss the relationship between protein and mRNAs, as well as proteogenomic approaches, *e.g.* (1–4), we will focus here on other new directions that have emerged in the last few years, *e.g.* with respect to combination of proteomics with other technologies or other data types. We will also discuss components of such integrative analysis.

### Integrating Proteomic and Transcriptomic Measurements

#### 

##### Correlating Protein and mRNA Concentrations

The first and long-debated question in integrative proteomic studies concerns the correlation between mRNA and protein concentrations in a steady-state system, *i.e.* in unperturbed cells that do not change over time. High correlation between mRNA and protein concentrations implies that transcription determines the cellular architecture; low correlation implies a dominant role for post-transcriptional regulation. For yeast and mammalian cells, estimates started to appear over ten years ago and differed considerably (5): although most studies agreed on substantial contribution of post-transcriptional regulation to the overall expression landscape (6, 7), some argued for a dominant role of transcription (8).

In 2012, we attempted to synthesize these findings into a common theme: transcription regulation might often act as an on-off switch, whereas translation and protein degradation fine-tune actual concentrations, like a rheostat (2). This two-step process attributes to the different response signals for different groups of genes (9). A 2015 study of bone-marrow derived dendritic cells exposed to lipopolysaccharide supported this view (10): indeed, most of the responses to the stimulus were initiated by RNA expression changes. In comparison, protein levels for housekeeping genes were also altered substantially when examining absolute molecule numbers. Because of the high protein concentrations, the resulting fold-changes remained comparatively small. We observed a similar trend in cancer cells responding to protein misfolding: protein concentration changes were much smaller in magnitude than mRNA expression changes (11). In addition, the transcriptome returned to pretreatment levels after ∼12 h whereas proteins did not reach steady state at that time. Our most recent study suggests that the cell might implement such discordance observed between the transcriptome and the proteome in different ways, *e.g.* through gene specific increase in translation via short regulatory elements despite no transcription change or an increase in transcription but delayed translation (12).

Another possible explanation for the disparity between transcript and protein levels has been discussed for yeast undergoing meiosis (13). When comparing protein expression levels with those of transcripts and parallel ribosome footprinting data, the authors noticed impaired translation of several genes through new isoforms which they named “long undecoded transcript isoforms” (LUTIs)^1^. They proposed that a single transcription factor can active the canonical transcript for some genes and LUTI for others.

Finally, several theoretical studies highlighted the unexpected conservation of protein-RNA ratios across tissues (14, 15), and the fact that protein concentrations of orthologs appear to be more conserved across organisms than mRNA concentrations (16–18). However, some of these observations are because of an effect like Simpson's paradox (19): some relationships may become reversed or masked by the opposing effects of other data types. Therefore, orthologous protein concentrations might be correlated across genes, but not as much as some studies suggest (20, 21).

In addition to these insights into the relationship between protein and mRNA concentrations, the future might bring more integration of these paired data sets with additional information, such as the temporal changes in response to a stimulus, physical interactions between proteins, or measurements of synthesis and turnover rates. Examples of such new directions include the time-resolved studies described above, or recent work involving Down Syndrome patients (22). The study analyzed protein and mRNA concentrations in samples from identical twins where one twin is healthy and one has Down Syndrome. Thanks to careful integration of these data with protein stability measurements, the authors demonstrated the major role of degradation in maintaining stoichiometry in protein complexes, despite minor effects on overall mRNA or protein levels.

##### Proteoforms and Alternative Splice Variants

Another factor lowering RNA and protein correlations arises from alternative splicing and the production of protein isoforms, and many efforts in integrative proteomics concern the complete mapping of the resulting proteome diversity (13, 15, 23–25). Integrating data from mRNA sequencing and proteomics, Liu *et al.* attempted to map the entire human proteome with respect to its variants (26). The authors identified a significant contribution of alternative splicing to proteome composition and diversity, with respect to alternative translation initiation, alternative splicing, and post-translational modifications. However, these estimates are not uncontended - other studies suggest that the number of functional variants per protein might be very small (27–29). The reason for these discrepancies may lie in technical challenges to identify critical peptides that mark variants and isoforms (29) or in the fact that most proteins get expressed only one isoform per tissue (23). Therefore, identification of functional proteoforms and alternative splice variants remains a daunting task.

### Combining Proteomics with other 'Omics Data

#### 

##### Measurements of Translation

Several recent studies have moved beyond simple assessment of the relationships between concentrations and toward identification of the underlying processes that determine concentrations and concentration changes. One example is the twin study mentioned above that included examination of protein degradation through use of dynamic proteomics (22).

Other examples arise from the inclusion of sequencing data that identifies ribosome footprint positions along mRNAs, estimating translation efficiency and regulatory elements (30). Several such studies exist and examined meiosis or the response to environmental stress (13, 15, 23–25, 31, 32). For example, when comparing genome-wide transcriptome, proteome, and ribosome profiles across diverse stresses, Ho *et al.* found that ribosomes appeared to dissociate from some transcripts, delaying their translation into the corresponding proteins (32). The authors suggested that this process frees ribosomes which could then be used toward the synthesis of stress response proteins.

However, importantly, the association of ribosomes with mRNAs may not always reflect actual translation output, as can be measured by proteomics methods such as pulsed SILAC (stable isotope labeling of amino acids) (33). Ribosomes might attach to mRNAs leading to reported footprints, but not actively translate and produce protein. Such discrepancy was observed for data from multiple myeloma cells (34). Under unperturbed conditions, ribosome footprinting and pulsed-SILAC translation measurements largely correlated across genes. However, when the cells were perturbed through inhibition of protein degradation, the correlation vanished: pulsed-SILAC was able to detect global alterations in translation rates across genes, whereas ribosome footprinting failed to do so. Therefore, albeit proteomics methods often pose technical challenges and result in smaller coverage than sequencing methods, some biological questions might demand the use of proteomics for translation measurements over assessment of ribosome footprints.

##### Genetic Association and Quantitative Trait Loci

An entirely orthogonal area of proteomic data integration lies in their use as molecular phenotypes that are then associated with specific genomic regions to discover Quantitative Trait Loci (QTL). Associations with mRNA expression phenotypes are typically called eQTLs, whereas associations with protein expression render pQTLs. The relationship between eQTLs and pQTLs is complex and still only incompletely understood (35, 36). For example, in yeast, the genomic position of eQTLs and pQTLs seems to overlap only little (37), and cis regulation is common for eQTLs but not at the level of the proteome (38). One important role of pQTLs appear to be maintenance of the stoichiometry of protein complexes and pathways (39).

More recent studies in blood plasma cells confirmed the complexity of the relationship between pQTLs and eQTLs. For example, they detected several pQTLs that affected protein levels in *trans*, illustrating how pQTLs can identify effects hidden at the mRNA level (40). Other studies found several pQTLs acting in *cis* and reported substantial overlap between pQTLs and cis-eQTLs—contrasting what had been observed before (41–43).

##### Post-translational Modifications

Protocols to measure post-translational modifications such as phosphorylation, ubiquitination, and SUMOylation are now readily available for routine use. Recent work integrated such measurements with other 'omics data, *i.e.* protein concentrations. For example, a study in mice showed substantial overlap between protein abundance and phosphorylation levels but revealed differences in the temporal patterns upon induction of a high-fat diet (44). A similar observation was made in samples from breast cancer patients: the phosphoproteome grouped into clusters that were undetectable at the mRNA or protein level, illustrating the need for collecting multiple data types (45).

However, it is crucial to move beyond simple parallel analysis to models that attempt to reveal and exploit relationships between the data (46, 47). Such analyses are necessary to understand causal relationships, *e.g.* the role of multiple, often successive protein modifications in signal transduction cascades. A step toward such analyses arises from a study of phosphorylation, acetylation, and methylation events across 45 untreated and treated lung cancer cell lines (48). Using machine learning and a comprehensive protein-protein interaction network, the authors found many multimodification events that acted in a mutually exclusive pattern: the protein had either one type of modification or the other, but not both. Such pattern suggests that protein modifications are used to direct signaling pathways into different routes with an “exclusive OR” gate depending on the type of modification.

##### Integrating Proteomics with Other Technologies

Another type of integrative developments uses mass spectrometry in conjunction with other technologies in form of a new method. Such analyses might not be strictly “integrative” yet, as they typically acquire only one data type. However, they might inspire and encourage proteomicists to venture more frequently into new territory to map novel aspects of biology.

For example, recent work combined proteomics with polysome profiling, a technique that exploits differential sedimentation of ribosome-bound mRNAs and the unbound the small and large subunits in a sucrose density gradient (49). The analyses examined mammalian cells during mitosis to identify changes in ribosome composition and function (50), as well as phosphorylation changes (51). Although the authors could not confirm earlier findings on varying composition of the ribosome core (52), they found extensive differential protein phosphorylation across the polysome profile leading to identification of a new regulatory phosphorylation event on a ribosome subunit.

Proteomics has also been combined with cellular thermal shift assays (CETSA) to monitor the thermal stability of the proteome (53). The method exploits the fact that, depending on their structural properties, proteins “melt” at higher temperatures and collapse (54). Integrating such stability measurements with estimates of protein abundance and solubility, the study found that many intrinsically disordered and mitotically phosphorylated proteins were stabilized and more solubilized during mitosis, suggesting a fundamental remodeling of the biophysical environment during the cell cycle. A more recent study using CETSA examined a similar system and found very little proteome remodeling during mitosis, but substantial changes in protein-protein interactions (55)—emphasizing the importance to look beyond simple concentration measurements for new discoveries.

A third expansion of traditional proteomics employs limited proteolytic digest prior to mass spectrometry, providing an indirect readout of a protein's structural stability through identification of peptides that are accessible to the protease (56, 57). Leuenberger *et al.* used this technique with samples processed at different temperatures to estimate thermal melting points of proteins from *Escherichia coli, Saccharomyces cerevisiae, Thermus thermophilus,* and human cells (58). Their results confirmed the complexity of the relationship between protein concentrations and stability and the need for a multifaceted analysis: although highly expressed proteins were particularly stable, a specific subset of proteins was destabilized by higher temperatures leading to cellular collapse.

##### Metabolomics and Lipid Measurements

Besides nucleic acids, the cell contains many other molecules with various functions—and proteomic data integration has made big strides toward understanding them. Such studies have, for example, combined limited proteolysis-based proteomics with screening of small molecules for their effect on protein folding (59). The authors found ∼140 new proteins that appeared to bind ATP, and many cases in which ATP or other metabolites affected the structure and protease accessibility of the protein. The role of ATP beyond being an energy source was also confirmed by use of CETSA in combination with proteomics: ATP-binding membrane proteins shifted in their sensitivity to detergents, suggesting that the molecule stabilizes the protein structure and increases solubility (60).

Other examples support this relationship between metabolism and the proteome. In Drosophila cells, measurements and calculations of phase differences revealed a link between oscillations of protein concentrations and their downstream metabolites (61). In other studies, integration of proteome, metabolome, and lipidome measurements revealed new functions of genes in mitochondrial coenzyme Q biosynthesis and regulation (62, 63). Finally, combining such measurements with genomic data defined lipid-QTLs like those discussed for mRNAs, protein, and translation discussed above. Indeed, lipid-QTLs differed substantially from protein QTLs as discovered by analysis of a BXD mouse population (64, 65). These findings were enabled through combination of multiple 'omics measurements, but analysis is currently restricted to mostly correlative observations. The next and exciting challenge lies in identifying causal relationships behind molecules and pathways.

### New Tools and Techniques for Integrative Analysis

#### 

##### General Tools

**Fig. 2.**
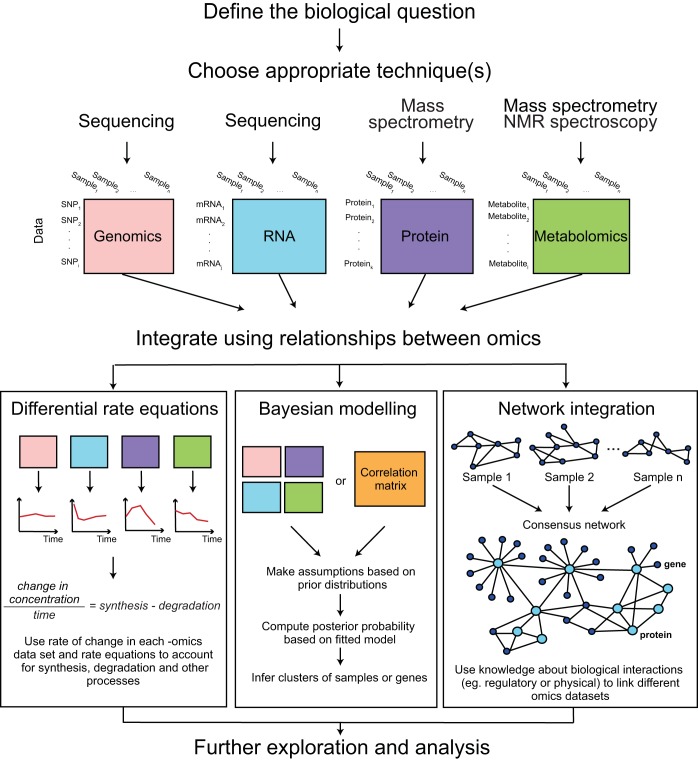
**Steps toward productive integromics.**

The growing demand for effective data integration has spurred the development of numerous computational tools and approaches. Several recent reviews comprehensively summarized these advances (66, 67). In addition, integrative tools have begun to emerge to model multiomics data including proteomics data (68). From a biologist's point of view, the major challenge in integrating multiple 'omics data may lie not in lacking availability, but rather in the selection of appropriate tools for a given research question. As the principal aims of the tools are diverse, ranging from the discovery of a sparse set of data features associated with a phenotype of interest to the integrated clustering of samples, finding the right tool can be demanding (Fig. 2). Therefore, no single computational tool can provide solutions for every problem, and successful application depends on the user's proper understanding of the biological question and the functionalities of each tool.

##### Visualization

An important starting point for successful integration is the visualization of integrated data to provide a holistic view of merged data. In the past, multiomics analyses typically used a series of heatmaps. However, heatmaps lose their merit with increasing data size, and they fail to show the connections between different types of molecules. Therefore, several tools have been designed to show networks of interfeature relationships. One example is the 3Omics tool in which biological networks are created based on a coexpression analysis of multiomics data and the resulting networks are displayed (69). More recently, OmicsNet has been developed to simultaneously map multiple 'omics data onto a network consisting of protein-protein interactions, metabolic relationships and co-expression information (70). The tool creates a global view of the network ensemble with flexible options for customization. Its visualization is unique as it not only preserves a modular network layout within each 'omics data but also shows the connections between different layers.

##### Data Interpretation

Another key to successful data integration is finding tools that can effectively harmonize the biological information across heterogeneous platforms and facilitate biological interpretation of merged findings. Many statistical methods already exist that can integrate multiple data sets to perform sample clustering or classification (71). In many cases, however, these tools treat different 'omics data as equally contributing features and ignore biological relationships between different types of molecules. For example, a recently published MultiOmics Factor Analysis tool (MOFA) is an unsupervised method for inference of a set of latent factors which can best capture sources of variabilities across the different 'omics data sets using a probabilistic Bayesian framework (72). However, as there may be no biological relationship between the features from different data types with high loading scores, the latent factors identified may not always necessarily be interpretable.

In contrast, computational methods that directly derive molecular interactions or communities of molecules from the correlation structures often yield more biological interpretable results. For example, a tool called Meta-dimensional Knowledge-driven Genomic Interactions (MKGI) maps molecular abundance measurements in each 'omics data set to the dimension of pathways and builds an ensemble of models to predict a phenotypic outcome via a neural network algorithm (73). Another tool called the OmicsIntegrator finds putative pathways using a network optimization algorithm and derives a subnetwork of the multiomics signature that best explains the expression data sets (74). Using a similar approach, the inteGREAT tool discovers co-expression networks within the transcriptome and the proteome and performs network analysis of differentially abundant genes, *e.g.* clinical biomarkers, incorporating network topology information for each gene from both 'omics levels (75).

##### Accounting for Specific Properties of Proteomic Data

Many integration tools treat proteomics data as if it is just another dimension in the 'omics repertoire, ignoring the fact that protein abundance and other quantitative features, *e.g.* post-translational modifications, are the ultimate output in gene expression regulation. Nevertheless, those tools do not necessarily inform on the underlying processes and determinants of the output, such as translation or degradation. Examining changes in protein numbers in a static condition does not necessarily stem from a shift in translation, as such a change could also be caused by altered protein degradation, localization or even formation of isoforms. Although some tools such as PARADIGM reflect the hierarchical nature and directionality among data from different 'omics platforms (76), incorporation of inter-omics relationships in statistical modeling has been mostly overlooked. In addition, parallel measurements of concentration changes and the underlying processes are still very rare, restricted to the few examples described above.

Further, as illustrated above for mammalian cells responding to an outside stimulus (10–12), gene expression control often results in large fold-changes in the transcriptome, but only small changes in the proteins. Such properties can skew joint analysis of the data in favor of the part of the data showing changes of a greater magnitude, *e.g.* mRNA expression. As such, integration of data across different experimental platforms requires appropriate normalization of data, *e.g.* transformation of data into scores on a standardized scale before performing joint analysis. Also, one can consider weighting the data sources with quality or informativity scores so that each 'omics data type can contribute to the analysis in equal proportions.

For some specific biological questions, such tools and techniques already exist and enable the user to normalize the data, estimate statistical parameters of interest, and interpret the results. For example, we and others have developed models to estimate protein synthesis and degradation from dynamic data, *e.g.* for the response to lipopolysaccharide or ER stress (10–12). In these cases, the relationship between the two paired data types, protein and mRNA concentrations, is a direct result of the central dogma of biology, and rate equations serve to quantify the parameters. The analyses provide precise estimates of significant regulatory events and time points.

We recently expanded this approach to provide a comprehensive tool to infer different regulatory parameters from any dual-omics time series experiment (77). The tool is called Protein Expression Control Analysis (PECA) and consists of autoregressive sub-models, each mimicking a first-order ordinary differential equation of abundance dynamics for a protein. The key integrative aspect of the approach comes from the incorporation of time series measurements and a flexible statistical method to estimate time-varying rate parameters with assessment of the noise in the data (false discovery rates). Indeed, we successfully used PECA to parse time-series transcriptomics measurements or even evaluate ribosome binding and dissociation with mRNA (12). In this case, significant changes in association of ribosomes with mRNAs as reported by PECA served as a readout for changes in translation of the respective gene. PECA is easily accessible as a plugin for the PERSEUS toolbox which allows for visualization, normalization, and interpretation of proteomics and other large-scale data (78).

### Future Directions: How to Exploit the Synergy of Productive Integrative Work

#### 

##### Steps Toward Productive Integromics

“Integromics” is an emerging form of proteomic analysis that exploits large-scale measurements of concentrations, modifications, and interactions of proteins with themselves and other molecules. The new challenges lie in experts from individual disciplines to become “multilingual” to understand the properties, information types, and limitations of data from other domains. Such multidirectional understanding is necessary to model the relationships between the data and exploit the information gained (Fig. 1).

What might be crucial steps toward such integrative work? As for all research, the first step should lie in defining the biological question, and all subsequent steps should always go back to this question (Fig. 2). As an integrative systems biologist, it will be important to step outside one's comfort zone and specific research field. True discovery might lie in exploration of new areas and techniques, such as the combination of proteomics with genetic information, the metabolome, the lipidome, translation measures or cellular melting, as discussed with the examples above.

The second step involves consideration as to what type of information is needed and what are the appropriate technologies (Fig. 2). If a question can be answered by studying just one molecular type, then there is no use in acquiring other data. For example, it is tempting to combine genome-wide analysis of RNA concentrations with the measurements of corresponding proteins. However, proteomics is typically more labor-intensive, assesses only a fraction of the expressed genome, and delivers noisier measurements. Therefore, mapping the proteome might only be worthwhile if there is evidence for regulation beyond what is captured by the transcriptome—warranting integrative analyses that involve multiple data types. For example, the response to protein misfolding stress is well-known to affect not only transcription, but also translation and RNA and protein degradation, therefore such integrative analyses are warranted.

Further, the choice of technique should involve consideration of the type of information to be gained. For example, proteomics techniques that provide absolute concentration estimates, such as parts-per-million or molecules per cell, are different from those estimating fold-changes in a specific condition *versus* a control. The former can be obtained by label-free quantitation or use of tags such as iTRAQ or TMT, the latter can be obtained from SILAC experiments. For example, when discussing the correlation between mRNA and protein concentrations, one needs to acquire absolute concentration estimates (1). When aiming to analyze rates of synthesis and degradation, time-resolved measurements of either absolute concentrations or fold-changes work, but the two different data types require different modeling (7, 10, 77).

In addition, the choice of adequate technique depends on the method's limitations. Proteomics is always biased toward peptides that are amenable to proteolytic digestion, solubility, and mass spectrometry. Therefore, a hunt for new splice variants may require use of alternative proteases to increase the proteome space. Further, ribosome footprinting provides genome-scale estimates of ribosome locations along mRNAs, but only indirect measures of translation efficiency. If the biological question asks for translation rates or evidence exist for extensive stalling of ribosomes without producing proteins, like for example during inhibition of the proteasome (79), ribosome footprinting may not be the method of choice.

A third step in integrative analyses lies in the careful planning of the experiment, keeping the anticipated statistical model in mind (Fig. 2). It should include considerations as to the number of genes that are involved and the number of biological or technical replicates to ensure enough statistical power. For multiomics experiments, it is important to ensure that the measurements are matched between techniques with respect to time points, conditions, and genes. If not, then how can the differences in experimental conditions be accounted for in the statistical model?

After data acquisition and normalization, the fourth step warrants the appropriate modeling of the relationship between the proteomics data and the other data types (Fig. 2). These relationships are the key to extracting information beyond what is gained from parallel analysis of individual data sets. As discussed above for tools, such modeling should include the hierarchical relationship between proteomic and other information, reflecting the proteins' position in the cellular system. Because “integromics” is a new field, there is no one type of recommendation yet to model such relationships; many different tools exist.

For example, in some cases the relationships can be expressed by rate equations, such as has been done for dynamic analyses of protein and RNA concentration changes in response to treatment with lipopolysaccharide or ER stressors (10, 12) (Fig. 2): changes in protein concentrations are expressed as a function of translation based on the mRNA concentration and protein degradation. In other cases, the relationships can be modeled in form of a network. An example of such studies examines oligodendrocytes in the context of Alzheimer's disease (80). Using transcriptomic and proteomic data, genome-wide association data, a protein-protein interaction network, and Bayesian analysis, the study identified Cnp as a main regulator that control the aspects of myelin and mitochondrial gene expression dysregulation. The relationship between proteins was modeled through known physical interactions that are part of signaling pathways.

A fifth and perhaps most enjoyable step of integrative analysis lies in letting go of all the restrictions mentioned above and play (Fig. 2). The benefit of large-scale studies lies in unbiased description of connections that can discover entirely unexpected and new patterns. Key to such exploration is visualization of the data in various ways, and several helpful techniques and tools are discussed above. Of course, an analysis should be guided with the original biological question in mind - but because the data commonly covers hundreds to thousands of genes, and because the relationships are usually based on general models and not manually curated information, it is quite possible to move beyond what is known and unravel new links. For example, in our own work, a large number of translationally upregulated genes during the ER stress response had been observed before, but after mapping these changes to different pathways of the mitochondrial energy metabolism, we discovered an entirely new trend: translation upregulation seemed to support a shift in energy metabolism from the tricarboxylic acid cycle to one-carbon metabolism (12).

##### Future Directions

Proteomic analysis and systems biology in general have exciting times ahead. We predict that future work will expand existing approaches in multiple ways (Fig. 1). Such studies might include actual experimental measurements of rates of transcription, translation, RNA and protein degradation all in parallel—to complement what has been modeled based on concentration measurements. Methods to measure these processes exist already, but their integration is still rare. Apart from computational models (81) or steady-state analysis (7), there is no study in which all four major synthesis and degradation rates have been measured simultaneously in one dynamic system. Results from such work would inform us on the relationship between the different processes, feedback and coupling between them, or different regulatory goals for different genes.

Future work might also involve more analyses of multiple modifications per protein or peptide—moving away from studying one post-translational modification at a time. When including temporal information, it will be possible to extract hypotheses on causal relationships, *e.g.* resolve which phosphorylation might trigger subsequent SUMOylation or ubiquitination on a specific protein. Such sequential modifications have been described for individual pathways, *e.g.* FEN1 (82) but are unknown with respect to the global cellular role.

Future integrative proteomics may also involve increased integration with other molecule types, such as those of the metabolome. Like the examples described above, such analyses will expand our knowledge of both binding sites in protein structures but also the impact of metabolites on protein stability and function (40, 54). To help such new analyses, we foresee more and more studies in which proteomics is combined with other technologies, such as the thermal profiling or polysome profiling mentioned above (52, 54).

And finally, future integrative proteomics might transcend into entirely new areas, *e.g.* the analysis of very small samples or single cells (83–87). Small sample analyses can reveal local relationships between cells in a tissue, as has been shown for HeLa cells (88). Such information could open many new applications ranging from analysis of rare cell populations to those of limited clinical specimens. Single cell analysis will also expand our views on the relationship between protein and mRNA concentrations (83), as has been discussed for bacteria many years ago (89).

In our view, the essential components to such integrative work will remain not only in combination of proteomics with other techniques and data types, but also the careful and quantitative modeling of the relationships between data as new findings lie in these relationships. Nothing in biology works in an isolated manner: protein changes are both impacted by various processes, but also affect multiple processes in the cell. The time is ripe to move toward the next level of systems biology and consider processes in combination, gaining new insights at their interface.

## Footnotes

^1^ The abbreviations used are:

LUTI: long undecoded transcript isoforms
SILAC: stable isotope labeling of amino acids
QTL: quantitative tract loci
CETSA: cellular thermal shift assays
MKGI: meta-dimensional knowledge-driven genomic interactions
PECA: protein expression control analysis.
